# Identifying Geographic Cold Spots of PCOS Diagnosis in Texas: A Spatial Analysis of Underdiagnosis and Rural Disparities

**DOI:** 10.1210/jendso/bvaf123

**Published:** 2025-08-04

**Authors:** Ryan Ramphul, Geethika Yalavarthy, Jooyeon Lee

**Affiliations:** Department of Epidemiology, UTHealth Houston–School of Public Health, Houston, TX 77030, USA; Department of Epidemiology, UTHealth Houston–School of Public Health, Houston, TX 77030, USA; Department of Biostatistics & Data Science, UTHealth Houston–School of Public Health, Houston, TX 77030, USA

**Keywords:** polycystic ovary syndrome, underdiagnosis, health disparities, rural health, geospatial analysis, Texas

## Abstract

**Context:**

Polycystic ovary syndrome (PCOS) is a common yet underdiagnosed endocrine disorder with substantial reproductive and metabolic consequences. Although disparities in PCOS care have been documented, few studies have employed spatial methods to identify areas of potential underdiagnosis.

**Objective:**

This study uses geospatial analysis to detect cold spots of PCOS clinical encounters across Texas and investigates neighborhood characteristics associated with these areas.

**Methods:**

We analyzed inpatient and outpatient encounter data from the Texas Public Use Data File (PUDF) between 2018 and 2024 to identify PCOS-related visits (International Classification of Diseases, revision 10: E28.2). ZIP code tabulation area (ZCTA)-level PCOS encounter prevalence was calculated per 1000 females and stabilized using empirical Bayes smoothing to account for rate instability. The Anselin local Moran's I statistic was used to detect spatial clusters. ZCTAs with statistically significant low-prevalence clusters (cold spots) were identified. Logistic regression assessed associations between cold spot status and neighborhood-level variables, including rural-urban commuting area codes, socioeconomic indicators, and health-related factors.

**Results:**

Cold spots were concentrated in rural and periurban areas, suggesting potential underdiagnosis in communities with limited health-care access. This highlights the need for targeted public health interventions, including expanded provider training and diagnostic outreach in rural settings.

**Conclusion:**

Significant spatial disparities in PCOS diagnosis suggest differential health-care access, diagnostic practices, or population health behaviors across the state. Targeted health interventions in rural communities may improve PCOS recognition and care. Further research is needed to explore the role of infrastructure and provider practices in causing these geographic disparities.

Polycystic ovary syndrome (PCOS) is one of the most common endocrine disorders affecting individuals of reproductive age, with a prevalence estimated at 5% to 15% globally, depending on diagnostic criteria [1]. Characterized by a constellation of symptoms, PCOS typically presents with irregular menstrual cycles, hyperandrogenism (eg, excess facial or body hair), and polycystic ovaries visible on ultrasound. Beyond reproductive health, PCOS is associated with a wide range of metabolic and psychological complications, including insulin resistance, type 2 diabetes, obesity, cardiovascular disease, anxiety, and depression [1].

While the exact etiology of PCOS remains unclear, it is believed to result from a complex interplay of genetic, hormonal, and environmental factors [2]. Early diagnosis and management are crucial, as untreated PCOS can lead to infertility and chronic metabolic disease [3]. Management often includes lifestyle changes, hormonal therapies, and tailored interventions to address specific symptoms and prevent long-term complications [1].

Despite the condition's high prevalence and substantial health implications, PCOS remains chronically underdiagnosed in many populations. Women of lower socioeconomic status and racial and ethnic minorities often face greater symptom burden and fewer opportunities for early detection due to structural barriers in the health-care system [4]. For example, an analysis of electronic health records from Boston Medical Center found that Black/African American women had significantly higher odds of missed PCOS diagnoses compared to non-Hispanic White women [5]. These disparities are further compounded by insurance-based inequities: Individuals with Medicaid or charity care coverage were nearly twice as likely to remain undiagnosed as those with private insurance [5].

Area-level indicators of disadvantage, such as the US Centers for Disease Control and Prevention (CDC)'s Social Vulnerability Index, are also associated with missed diagnoses, pointing to the role of neighborhood context in shaping PCOS detection and care access [5]. Together, these findings highlight an urgent need to understand where underdiagnosis is occurring—not only to improve disease surveillance, but also to better target outreach, provider education, and diagnostic support in underserved areas.

A major challenge in PCOS research is the absence of robust surveillance systems to monitor its true prevalence. Unlike many chronic conditions, PCOS lacks standardized diagnostic tracking, leading to sparse data and an incomplete picture of its geographic distribution [5]. While some studies have explored regional and racial disparities in PCOS prevalence, few have employed geospatial methods to detect areas where women may be systematically missed or underserved [6, 7].

This study addresses that gap by using the Texas Public Use Data File (PUDF), a statewide clinical encounter data set, to identify ZIP code tabulation areas (ZCTAs) where the prevalence of diagnosed PCOS is statistically lower than expected based on population. Cold spots, or areas with significantly fewer diagnosed PCOS encounters than expected, may reflect barriers to care, lower diagnostic awareness among providers, or structural inequities that suppress detection.

By examining neighborhood-level factors associated with these cold spots—including rurality, socioeconomic indicators, and demographic composition—this analysis offers a novel contribution to the PCOS disparities literature. The findings are intended to inform public health strategies for improving PCOS recognition and care equity in underserved and overlooked communities.

## Materials and Methods

### Data and Data Processing

Data for this analysis were sourced from inpatient and outpatient encounter data contained in the PUDF, provided by the Center for Health Care Data at the University of Texas Health Science Center at Houston School of Public Health. The PUDF, managed by the Texas Department of State Health Services, is a valuable administrative data set that includes deidentified health-care encounter data from hospitals and other health-care facilities statewide. While not without limitations, the PUDF provides a unique opportunity to estimate PCOS burden at a granular level, offering insights that would otherwise be difficult to obtain given the challenges in PCOS surveillance at the neighborhood level.

We queried the PUDF for any inpatient and outpatient encounters with a diagnosis of PCOS (International Classification of Diseases, revision 10 code E28.2) anywhere in the discharge diagnosis field, between January 1, 2018, and July 30, 2024. Extracted variables included patient demographics (age, race, ethnicity, and ZIP code), visit-specific details (such as type of visit—emergency department, inpatient, or outpatient), length of stay for inpatient visits, and associated clinical data. Table 1 describes the demographic distribution of the clinical data. Clinical data fields captured diagnosis codes, comorbidities, surgical procedure codes (if surgeries were conducted during the visit), and additional health conditions reported during the encounter. The protocol has been approved by the Committee for Human Subject Research of the University of Texas Health Science Center at Houston (institutional review board No. HSC-SPH-24-0499).

**Table 1.** Age, race, and ethnicity distribution of individuals with polycystic ovary syndrome encounters in Texas, 2018 to 2024

Category	Subcategory	Count (%)
**Age, y**	<10	10 (0.0115%)
	10-19	4885 (5.60%)
	20-29	29 417 (33.7%)
	30-39	35 892 (41.1%)
	40-49	13 128 (15.0%)
	50-59	2946 (3.38%)
	60-69	728 (0.834%)
	70-79	211(0.242%)
	80-84	25 (0.0287%)
	≥85	17 (0.0195%)
**Race**	American Indian/Alaskan Native/Eskimo	293 (0.34%)
	Asian or Pacific Islander	2638 (3.02%)
	Black	11 228 (12.9%)
	White	61 392 (70.4%)
	Other	11 702 (13.4%)
	Invalid	6 (0.007%)
**Ethnicity**	Hispanic origin	24 832 (28.5%)
	Not of Hispanic origin	62 319 (71.4%)
	Invalid	108 (0.124%)

To estimate PCOS encounter prevalence at the neighborhood level, we created a ZCTA-level ratio by dividing the number of health-care encounters involving any mention of PCOS in the encounter data by the total number of females residing in each ZCTA, obtained from 2022 US Census Bureau's American Community Survey 5-year estimates. To account for potential instability in rates due to small population denominators, we applied empirical Bayes (EB) smoothing, which adjusts observed rates toward the statewide mean based on each ZCTA's population size. This method reduces the influence of random variation in sparsely populated areas and enhances the stability of rates used in cluster detection.

This approach provides a population-adjusted metric of PCOS-related health-care utilization, offering a meaningful proxy for PCOS burden across different geographic areas Although this method cannot account for undiagnosed or untreated cases, it represents a considerable advancement in understanding the spatial distribution of PCOS at a fine geographic scale.

### Statistical Analysis

We applied the Anselin local Moran's I statistic [8] to the Empirical Bayes–smoothed PCOS encounter prevalence per 1000 females to detect spatial clusters. This technique identifies local patterns of spatial association, including cold spots (ZCTAs with significantly low PCOS encounter prevalence surrounded by similarly low ZCTAs) and hot spots (ZCTAs with significantly high PCOS encounter prevalence surrounded by similarly high ZCTAs). Spatial relationships between ZCTAs were defined using Queen contiguity, and statistical significance was assessed using 9999 permutations. A false discovery rate correction was applied to account for multiple comparisons, and ZCTAs with adjusted *P* values less than .05 were classified as statistically significant clusters.

Following identification of statistically significant cold spots, we used logistic regression to examine ZCTA-level demographic and socioeconomic factors associated with cold spot status. Independent variables included indicators known to be associated with health-care access or chronic disease disparities, as detailed in Table 2.

**Table 2.** ZIP code tabulation area–level demographic, socioeconomic, and health variables used in cold spot analysis

Variable	Description
Food insecurity	Prevalence of food insecurity in past 12 mo among adults
Food stamps	Prevalence of receiving food stamps (SNAP) in past 12 mo among adults
Household income	Mean income in past 12 mo in dollars
Prevalence of high cholesterol	Prevalence of high cholesterol among adults
Prevalence of obesity	Prevalence of obesity among adults
Primary RUCA code	Code indicating urbanicity (RUCA 1) to rurality (RUCA 10). Codes are classified ranging from 0 to 10
Proportion of below-poverty level	Proportion of females below poverty level
Proportion of Hispanic	Proportion of Hispanic female population among total female population
Proportion of Non-Hispanic American Indian	Proportion of Non-Hispanic American Indian female population among total female population
Proportion of Non-Hispanic Asian	Proportion of Non-Hispanic Asian female population among total female population
Proportion of Non-Hispanic Black	Proportion of Non-Hispanic Black female population among total female population
Proportion of Non-Hispanic Other	Proportion of Non-Hispanic other races female population among total female population
Proportion of Non-Hispanic White	Proportion of Non-Hispanic White female population among total female population

**Abbreviations:** RUCA, rural-urban commuting area; SNAP, Supplemental Nutrition Assistance Program.

Race and ethnicity were included as proxies for structural and systemic inequities, rather than biological attributes, consistent with epidemiological best practices [9]. Including racial and ethnic composition at the ZCTA level helps reveal how contextual social disparities may contribute to geographic variation in PCOS diagnosis patterns.

We included socioeconomic indicators such as the percentage of residents living below the federal poverty line and median household income, obtained from the 2022 American Community Survey 5-year estimates. To reflect food access and nutritional security, we used 2023 ZCTA-level data from the CDC's PLACES project [10], including prevalence of food insecurity and SNAP (Supplemental Nutrition Assistance Program) enrollment. Also from PLACES, we included crude prevalence rates of obesity and high cholesterol—key metabolic risk factors associated with PCOS.

To assess urban-rural differences, we used the 2010 ZCTA-level rural–urban commuting area (RUCA) codes, which categorize areas from urban cores (RUCA 1) to remote rural areas (RUCA 10), based on commuting and population density patterns. These codes provide context for differences in health-care infrastructure, service availability, and diagnostic access across the state.

Prior to modeling, we excluded highly correlated variables (correlation ≥ 0.7) and applied log(x + 1) transformations to highly skewed predictors. All continuous variables (excluding RUCA) were standardized to *z* scores. We assessed multicollinearity using variance inflation factors to ensure model stability. Cold spot classification was derived from ArcGIS Pro, and all logistic regression analyses were performed in R version 4.2.2.

## Results

**Figure 1.**
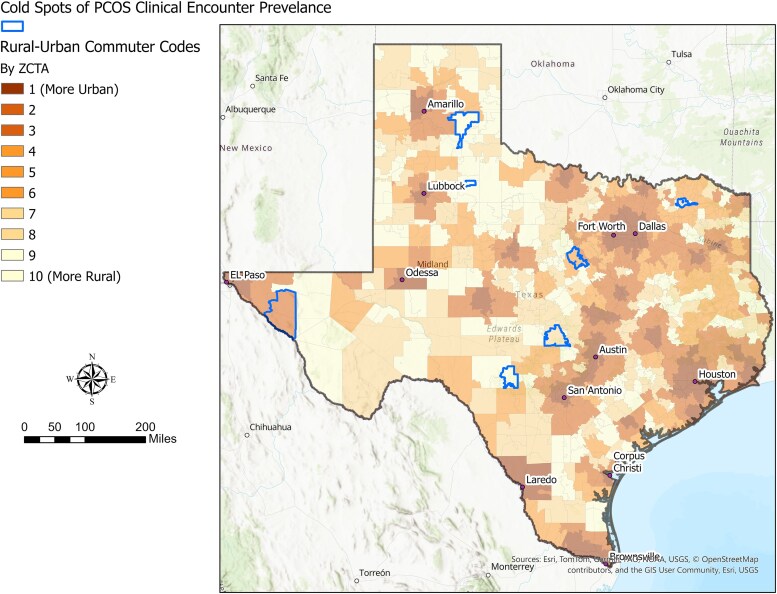
Statistically significant cold spots of polycystic ovary syndrome clinical encounters in relation to rural-urban commuting area codes in Texas.

Using empirical Bayes–smoothed encounter rates, we identified 7 ZCTAs in Texas as statistically significant cold spots for PCOS clinical encounter prevalence, based on the Anselin local Moran's I statistic. The remaining 1791 ZCTAs were classified as noncold spots. Figure 1 displays the geographic distribution of cold spots in relation to RUCA codes. Notably, most cold spots were located on the periphery of major metropolitan areas, suggesting proximity to urban cores but potential gaps in health-care access or utilization.

In preparation for multivariable logistic regression, several neighborhood-level variables were excluded due to data limitations. Household income was removed due to missingness, which would have substantially reduced the number of observations. Variables for the proportion of non-Hispanic White residents and food insecurity were excluded due to high collinearity with other covariates. Additionally, the obesity prevalence variable was removed based on elevated variance inflation factors (> 5), ensuring multicollinearity did not compromise model stability.

The final regression model evaluated the association between cold spot status and ZCTA-level factors. RUCA code was the only statistically significant predictor. A one-unit increase in RUCA code—indicating a shift toward a more rural classification—was associated with 33.9% higher odds of a ZCTA being a cold spot for PCOS encounters (odds ratio = 1.339; 95% CI, 1.048-1.783; *P* < .05). Table 3 presents the full regression results.

**Table 3.** Multivariable logistic regression results: ZIP code tabulation area–level predictors of cold spot status for polycystic ovary syndrome encounter prevalence

Variable	Odds ratio (95% CI)	*P*
(Intercept)	**0.001** (**0.000-0.003)**	**<**.**0001**
Proportion of Hispanic	1.107 (0.356-2.915)	.847
Proportion of Non-Hispanic Black	0.872 (0.159-2.323)	.832
Proportion of Non-Hispanic American Indian	0.619 (0.066-1.417)	.534
Proportion of Non-Hispanic Asian	1.171 (0.229-2.247)	.746
Proportion of Non-Hispanic other	0.906 (0.271-1.683)	.814
Proportion of below-poverty level	0.809 (0.296-1.584)	.646
Food stamp	1.230 (0.317-3.777)	.741
Prevalence of high cholesterol	0.952 (0.570-2.899)	.890
Primary RUCA code	**1.339** (**1.048-1.783)**	.**026**

Bold values indicate statistically significant predictors (*P* < .05). Intercept is significant at *P* < .001.

Abbreviation: RUCA, rural-urban commuting area.

## Discussion

This study highlights geographic disparities in the diagnosis of PCOS across Texas and identifies specific areas—referred to as cold spots—where significantly fewer PCOS clinical encounters are documented than would be expected given population size. These cold spots were more likely to occur in rural or periurban areas, as indicated by higher RUCA codes, reinforcing the importance of place-based determinants in PCOS recognition and care.

Unlike previous studies that have focused on areas with high disease burden (hot spots), our analysis emphasizes the underdiagnosis of PCOS. The identification of cold spots has important public health implications: These areas may reflect structural barriers to care, including limited availability of reproductive endocrinologists or primary care providers with PCOS knowledge, lower patient awareness, or reduced diagnostic vigilance among providers. This shift in focus aligns with broader efforts in health equity research to identify and intervene in places where care is not reaching those in need.

Our findings are consistent with prior literature documenting how rurality correlates with reduced access to specialized health care, and with research showing that systemic barriers—such as lack of insurance, transportation, or diagnostic infrastructure—contribute to delayed or missed PCOS diagnoses. For example, a study at Boston Medical Center found that women with Medicaid or charity insurance had nearly double the odds of a missed diagnosis compared to women with private insurance [5]. These disparities are not unique to PCOS; they mirror patterns seen in chronic disease management more broadly, such as with diabetes and hypertension.

Although race/ethnicity at the ZCTA level was not a statistically significant predictor in our model, this does not imply that racial disparities in PCOS diagnosis do not exist. Rather, it reflects a limitation of ecological-level analyses: Neighborhood averages can obscure individual-level patterns. Prior studies have shown that Black and Hispanic women are often underdiagnosed despite meeting clinical criteria for PCOS, likely due to provider biases, communication barriers, and structural racism embedded in health-care systems [11]. It is possible that the effects of race are mediated by or interact with rurality, insurance coverage, or provider availability—factors that warrant further investigation with more granular data.

This study has limitations. Most notably, our estimates are based on diagnosed cases of PCOS using International Classification of Diseases, revision 10 codes, and therefore do not capture the full prevalence of PCOS, particularly among women who remain undiagnosed. Moreover, the use of ZCTAs, while practical for linking to neighborhood-level data, masks within-area heterogeneity. One part of a ZCTA may differ substantially from another in terms of resources, population demographics, or environmental stressors [12]. Future studies using census tracts, patient-level data, or algorithmically defined PCOS cases could help to address these gaps.

Despite these limitations, our study provides a novel spatial analysis of PCOS diagnosis patterns across Texas, contributing to the limited literature on geographic disparities in women's endocrine health. By identifying areas with systematically low diagnostic activity, we offer a road map for targeted interventions. Public health efforts in these regions should focus on improving awareness of PCOS symptoms, expanding screening and diagnostic capabilities, and training rural providers on updated diagnostic guidelines. Investments in telemedicine, community health outreach, and policy supports for rural health infrastructure may be critical for reducing geographic disparities in PCOS care.

## Data Availability

Raw data were generated at the PUDF, provided by the Center for Health Care Data at the University of Texas Health Science Center at Houston School of Public Health. Derived data supporting the findings of this study are available from the corresponding author on request.

## Abbreviations

CDC: US Centers for Disease Control and Prevention
PCOS: polycystic ovary syndrome
PUDF: Texas Public Use Data File
RUCA: rural-urban commuting area
ZCTA: ZIP code tabulation area
